# Frac Sand Mines Are Preferentially Sited in Unzoned Rural Areas

**DOI:** 10.1371/journal.pone.0131386

**Published:** 2015-07-02

**Authors:** Christina Locke

**Affiliations:** Department of Forest and Wildlife Ecology, University of Wisconsin-Madison, Madison, Wisconsin, United States of America; Peking UIniversity, CHINA

## Abstract

Shifting markets can cause unexpected, stochastic changes in rural landscapes that may take local communities by surprise. Preferential siting of new industrial facilities in poor areas or in areas with few regulatory restrictions can have implications for environmental sustainability, human health, and social justice. This study focuses on frac sand mining—the mining of high-quality silica sand used in hydraulic fracturing processes for gas and oil extraction. Frac sand mining gained prominence in the 2000s in the upper midwestern United States where nonmetallic mining is regulated primarily by local zoning. I asked whether frac sand mines were more commonly sited in rural townships without formal zoning regulations or planning processes than in those that undertook zoning and planning before the frac sand boom. I also asked if mine prevalence was correlated with socioeconomic differences across townships. After creating a probability surface to map areas most suitable for frac sand mine occurrence, I developed neutral landscape models from which to compare actual mine distributions in zoned and unzoned areas at three different spatial extents. Mines were significantly clustered in unzoned jurisdictions at the statewide level and in 7 of the 8 counties with at least three frac sand mines and some unzoned land. Subsequent regression analyses showed mine prevalence to be uncorrelated with land value, tax rate, or per capita income, but correlated with remoteness and zoning. The predicted mine count in unzoned townships was over two times higher than that in zoned townships. However, the county with the most mines by far was under a county zoning ordinance, perhaps indicating industry preferences for locations with clear, homogenous rules over patchwork regulation. Rural communities can use the case of frac sand mining as motivation to discuss and plan for sudden land-use predicaments, rather than wait to grapple with unfamiliar legal processes during a period of intense conflict.

## Introduction

The siting of locally undesirable land uses (LULUs) like mines, waste facilities, power plants, prisons and feedlots depends on the actions of both industry and local communities [1–3]. LULUs can present a dilemma for rural communities: the potential of increased revenues and job growth on the one hand [3,4] and increased danger, ill health or lowered property values on the other [2,5]. In many parts of North America and Europe, it is the prerogative of local governments to regulate—or to not regulate—industrial and other land uses through zoning [6]. Many rural communities manage land-use conflicts informally, among neighbors and on an ad-hoc basis, and may be unfamiliar with formal planning and zoning processes [7]. Gradual changes like housing development, a growing commuter population, and other signs of suburbanization often prompt growing communities to adopt comprehensive land-use plans and zoning ordinances as reactive measures [7,8]. However, new and drastic changes may arise even in rural areas that show few signs of suburbanization and are outside of commuting distance to major urban centers. In these cases governments may need to navigate unfamiliar legal processes during a period of perceived crisis, such as a boom of extractive industrial operations. Informal agreements may be insufficient means for controlling development when facing new or stochastic market forces, even in slow- or no-growth rural areas.

This study focuses on a specific type of LULU, the mining of silica sand for use in the hydraulic fracturing industry (“frac sand mining”). The research objectives in this study are first to describe and analyze the spatial pattern of frac sand mines, and secondly to explore relationships between mine prevalence and socioeconomic variables that might explain why these patterns exist. In Wisconsin, USA where most high quality silica sand is located, frac sand mine siting and operations are regulated primarily through local zoning. However, many rural jurisdictions have no zoning or other formal land-use regulations. County officials report that mining companies locate preferentially in unzoned areas (Wisconsin county official, personal communication) but this assertion has not yet been tested formally. Following a body of research contending that industrial facility location depends on regulatory stringency [9–13], I hypothesize that a lack of zoning may provide a “path of least political resistance” for frac sand mine siting [14], and I expect to find more mines in jurisdictions without formal zoning regulations than those with preexisting zoning ordinances. I also investigate zoning stringency and socioeconomic factors hypothesized to influence industrial siting [5] to determine if these are more important predictors of frac sand mine prevalence than presence/absence of zoning. Unlike other LULUs like waste facilities or power plants, mines are limited to specific pieces of land where the resource to be mined is accessible. I therefore consider spatial variation in resource availability as a key component in the analyses.

## Background

### Factors influencing industry facility siting choices

The influences of transportation and production costs on siting are well established in the firm location literature, while the effects of sociopolitical factors are more contentious [13,15]. To minimize transportation costs between input and output markets, firms preferentially locate where there exits appropriate transportation infrastructure like highways and rail lines [16]. Production costs include the costs of acquiring labor and raw materials of sufficient quality. Firms are generally more likely to locate where education levels are higher [10,16] and wage rates are lower [17–19]. Industries that require specific raw materials or specialized labor forces tend to agglomerate where those resources are abundant [20–23]. In the case of extractive industries, facility location is constrained by the presence of the raw material to be mined. The rarer and more localized the mined resource, the higher the expectation of mine aggregation.

The extent to which sociopolitical factors influence firm siting is heavily debated and especially contentious for polluting industries and other LULUs. For example, the effect of taxes is not apparent across industries [16,19], but may be a more important factor for highly polluting industries than nonpolluting industries [10]. The “pollution haven” hypothesis predicts industrial facilities will be sited preferentially in jurisdictions where environmental regulations are less strict and present lower compliance costs for firms [9,13,15]. Though surveyed industry representatives assert that regulatory climate affects facility site choice [24], empirical testing of this hypothesis at international, national, and subnational levels have produced mixed results [10–13,17,18,25,26]. One distinction between studies showing evidence of a pollution haven effect and those that do not is spatial scale [13,15]. Comparing different countries or states washes out local variation that may matter more than country or state differences. Indeed, county-level studies have shown a larger pollution haven effect than studies at more aggregate levels [13].

Another contested hypothesis states that remote, disenfranchised communities are both more likely to pursue and be pursued by LULUs than more populated, wealthier areas [5]. It is common for communities experiencing industrial decline to seek opportunities for economic development even if it comes in the form of polluting industries [3], though scant evidence suggests localities compete for plants by lowering environmental standards [27–29]. From the point of view of industry, location choice is more efficient if the land is zoned for industrial uses and appraised at lower cost, areas that tend to house predominantly low-income and minority residents [30]. Hazardous waste facilities have been shown to locate where racial and socioeconomic disparities are greatest [14], raising issues of environmental justice and disproportional environmental costs to certain demographic groups. Theory posits that polluting plants will preferentially locate where communities lack political power and the likelihood of collective action in opposition to the plant is low [31,32]. It is unclear how these relationships play out in the case of frac sand mining which is constrained to a region lacking drastic disparities in race but with moderate variation in remoteness and wealth.

The above literature on industrial facility siting informed the choice of variables included in this study. Location costs of frac sand mining are expected to be lowest where there exists high-quality, easily accessible silica sand close to transportation infrastructure (major roads and rail lines). Policy barriers to mining are expected to be lowest in jurisdictions that have not adopted land-use plans and zoning regulations, the latter being the primary regulatory control of nonmetallic mine siting in Wisconsin. To determine if socioeconomics account for mine prevalence above and beyond what is predicted by silica sand availability and zoning regulation, I also consider the importance of township-level variables for income, race, tax rates, land values, parcel size, and remoteness.

The study subject requires analysis at the sub-county level, a finer spatial scale than any previous pollution haven work. A local focus is important because there is more variation in nonmetallic mining regulation at the local level than there is at the state level. State and federal rules that apply to all Wisconsin frac sand mine operators include having a reclamation plan before mining, and abiding by stormwater discharge, high capacity well, blasting, and air quality standards. Federal air regulations apply equally to all counties; no Wisconsin county containing silica sand has been found to be in noncompliance of federal air quality standards for silica dust [33]. Governing the location, size, and hours of operations, traffic and other issues is the purview of counties or municipalities and outside of state discretion [34]. Because frac sand mine location is regulated at the local level, an analysis ignoring within-county variation in zoning policy would ignore any potential siting preferences for local non-regulation.

### Hydrofracking and frac sand mining

The latter half of the 20^th^ century saw technological improvements allowing the creation of previously inaccessible oil and natural gas in horizontal shale deposits. Hydraulic fracturing (“hydrofracking” or “fracking”) is an established technique in the U.S. and Canada and under development in China and Europe [35]. During the hydrofracking process, water, chemicals, and a suspended “proppant” are driven deep into the earth under pressures high enough to fracture rocks. When the pressure is released, the proppant prevents the fissures from closing, allowing for the release of oil or gas from the newly opened mine. Proppants can be made synthetically from aluminum or ceramic beads, but because it is easily accessible domestically, naturally occurring silica sand remains the standard proppant used for hydrofracking in the U.S. [36].

Frac sand mining activity is limited to three sandstone deposits of the U.S.—the Cambrian Jordan Sandstone in Minnesota, the Cambrian Hickory Sandstone in Texas, and the Ordovician St. Peter Sandstone, covering western and central Wisconsin where the largest expanses of shallow silica sand deposits occur [36]. Sand suitable for use in hydrofracking is geologically old, deposited during the Cambrian and Ordovician periods of the Paleozoic Era when the Midwestern U.S. was covered by a shallow sea [34]. This ancient sea deposited many layers of pure quartzite beach sand at its edges as it grew and retreated over time. This sand is characteristic for its purity and is composed of greater than 95% silica quartzite. It is loosely packed and contains consistently sized, spherical grains. These qualities make frac sand resistant to erosion and the compressive forces required by the hydrofracking process.

**Fig 1:**
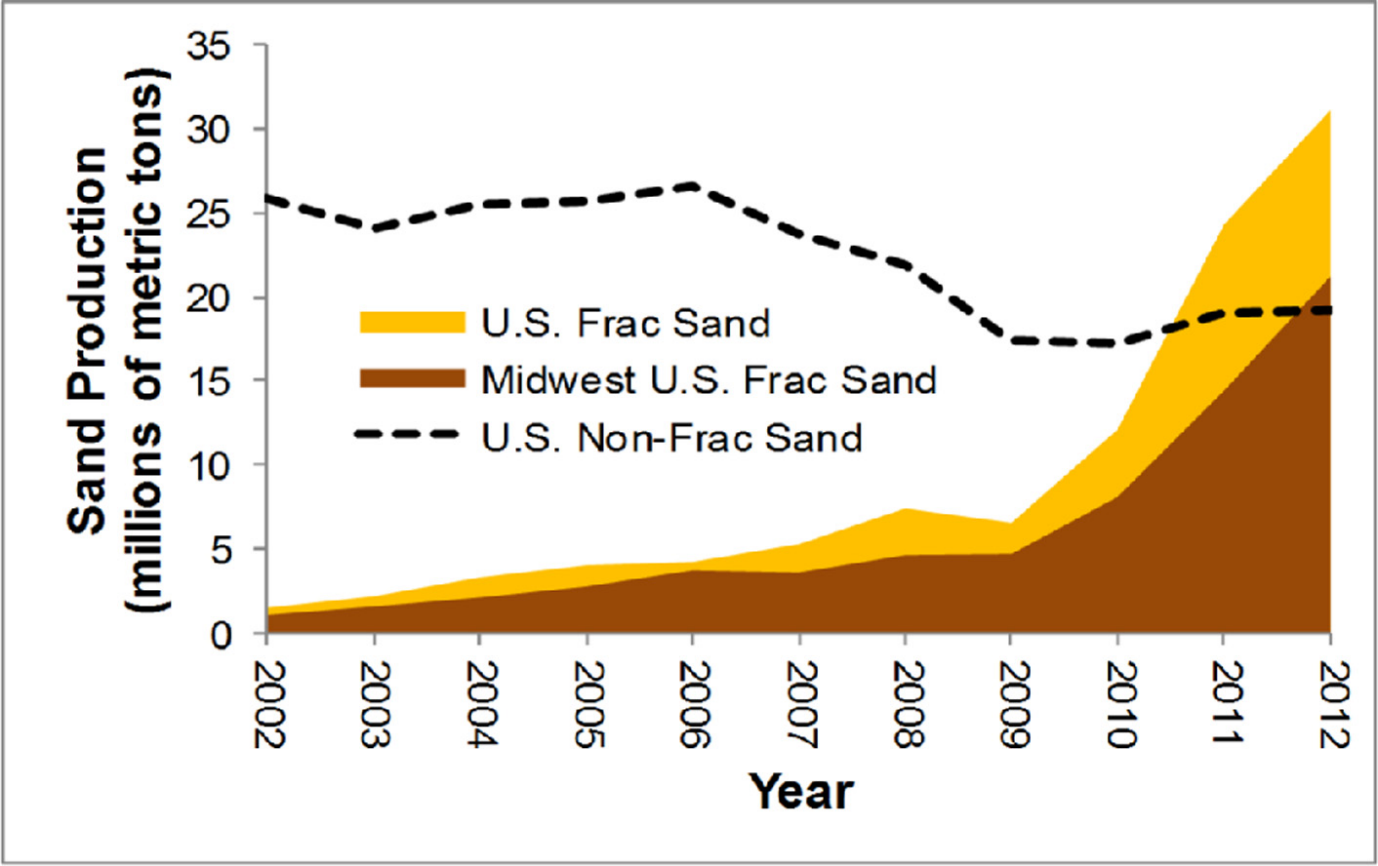
The exponential increase in U.S. frac sand production in the late 2000s. The Midwest accounted for 68% of U.S. frac sand production in 2012, with Wisconsin accounting for the largest overall share. Texas was the leading frac sand producer outside of the Midwest. The dotted line indicates U.S. sand production for all end uses other than hydraulic fracturing. Data: U.S. Geological Survey [36].

Silica sand has many industrial uses from glassmaking to filtration and abrasives, but the rapid increase in production after 2009 was almost entirely driven by a boom in the hydrofracking industry (Fig 1) [36]. In the three years between 2009 and 2012, U.S. production of frac sand increased by 85%, 101%, and 28%, respectively, to a total of over 30 million tons [36]. Frac sand mines operate on a larger scale than traditional sand mines, some covering over 150 ha and operating 24 hours a day for 9 months of the year [37].

### Benefits and consequences of extractive industries in rural communities

Localities aim to attract businesses and companies that will provide jobs and contribute to the local tax base [3,38], but even local governments looking to attract growth have reasons to be skeptical of mining. Though mining provides higher wages and employment than other extractive industries (agriculture, fishing and forestry), it is notoriously a “boom and bust” enterprise [39]. Communities dependent on specific extractive industries are at the mercy of market fluctuations, with rising costs and falling commodity prices often leading to economic decline after an initial period of prosperity [40]. Long-term economic effects of mining vary regionally. For example, coal mining in the 1980s and 1990s proved economically disastrous in the U.S. South and Great Lakes regions but economically prosperous in the West [41].

In the state of Wisconsin, where most frac sand mines are located, the frac sand mining industry is expected to directly add a total of 2300–2800 jobs [42], many of these truck driving jobs for sand transport. This amount of job growth might be called modest—it is about the same as the average monthly rate of job growth statewide between 1990 and 2010 [42]—but a promise of up to 40 new jobs from a single mine may be considered a huge boon to a struggling rural economy. Wisconsin has no statewide regulations for nonmetallic mine siting or operations [43], and it is up to local communities to determine whether a promise of new (and not necessarily local [42]) jobs is worth the environmental impacts and potential health risks associated with frac sand mining. Frac sand mining detractors are concerned about dangerous levels of silica particulates in the air, compromised groundwater quantity and quality, noise and costly road damage due to 24 hour-per-day truck activity, lowered property values in the vicinity of the mines, loss of soil integrity and farmable land, and loss of topographic variability in the landscape as mining levels sandstone bluffs [37]. Health risks associated with frac sand mining, in particular fine silica particulate dust and groundwater pollution from processing, are so far unclear, but several studies are underway [44–46]. In 2012 nearly one-fifth of Wisconsin frac sand operations were found in violation of statewide standards for air quality, stormwater runoff, or drillhole abandonment [47].

## Methods

### Study region

This study focuses on Wisconsin, USA, a state recognized as the frac sand industry’s “global epicenter” [48]. Wisconsin produces an estimated 28 million tons of frac sand per year, more than all other U.S. states combined [36,49]. Wisconsin’s silica sand deposits are extensive enough to produce up to 70 million tons of frac sand, double the projected total U.S. market demand for 2015 [49]. Many of west-central Wisconsin’s silica sand deposits are shallowly buried at depths less than 15.25 m, making extraction relatively easy [50]. Because Wisconsin does not have shale-oil deposits required for hydrofracking, all of its extracted frac sand is exported out of state. Importantly, western Wisconsin has extensive rail and road infrastructure in place allowing for bulk transport from frac sand mines to processing plants (where the sand is washed and sorted by grain size) and to railroad shipping stations for export to hydrofracking sites [36].

Siting and operations of frac sand mines in Wisconsin are regulated primarily through local zoning regulations [43]. Both counties and municipalities have authority to zone under Wisconsin’s zoning enabling legislation, but zoning is not mandated. Rural areas that are not incorporated as cities or villages may be zoned either at the township or county level, or they may remain unzoned. As of 2011, 20% of the 1255 townships in the state had not adopted a zoning ordinance, 19% were zoned at the township level, and 61% were zoned at the county level [51]. For the municipalities and counties choosing to regulate land use, a Wisconsin state law, effective 2010, requires a government’s regulatory land-use actions to be consistent with a comprehensive land-use plan. Over 90% of the municipalities required to plan by 2010 did so [52], and 131 of 246 unzoned townships elected to undergo a land-use planning process even though they were not required to under the law [53].

### Mapping frac sand mines

**Fig 2:**
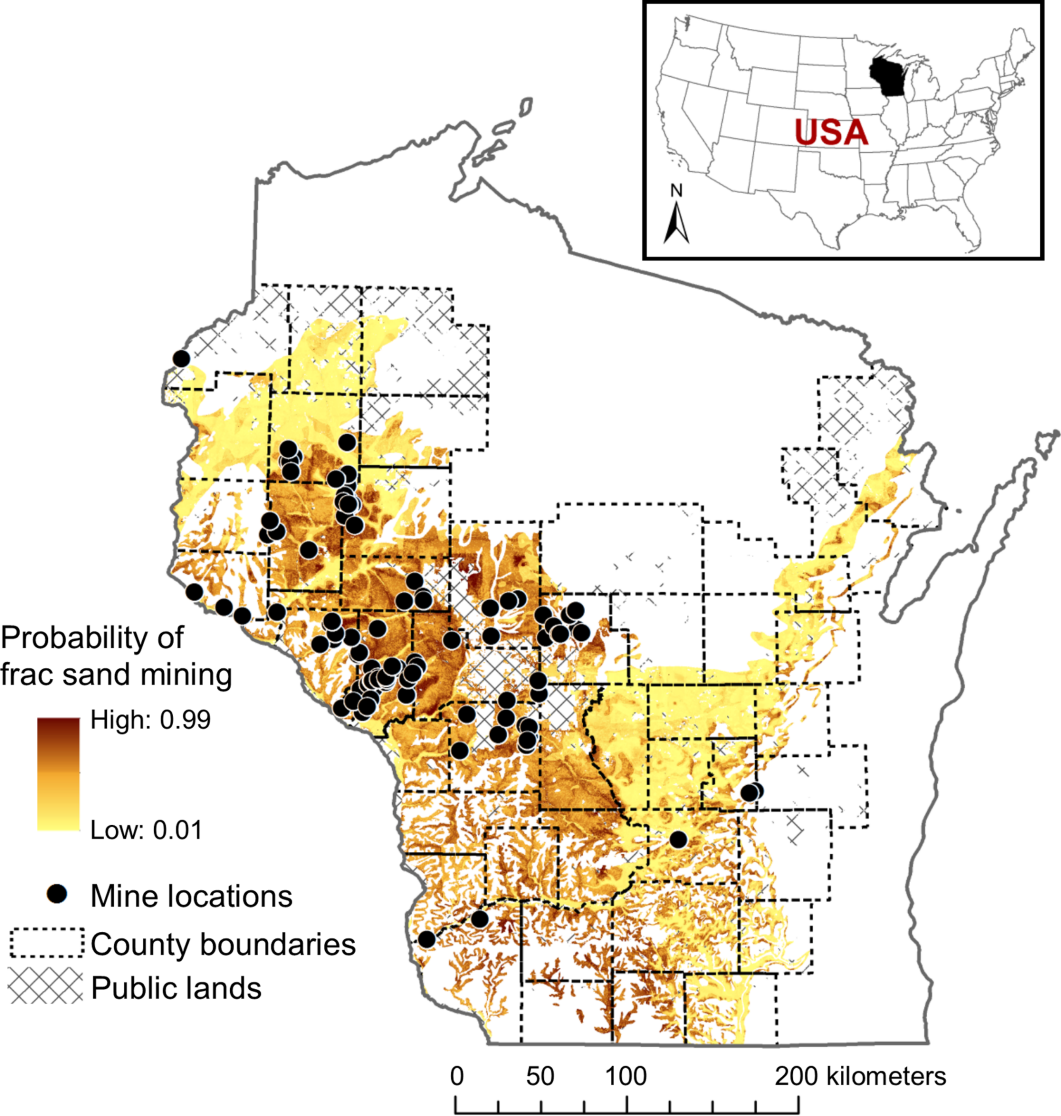
Map of silica sand deposits and probability of frac sand mine occurrence in Wisconsin, USA. Probabilities were calculated using Maxent software [57] based on depth to sand, distance to major roads and rail lines, and land cover type. County boundaries and public lands are shown only for counties containing silica sand.

I mapped 102 locations for all known frac sand mines in Wisconsin permitted, in development, or operational as of October 2013 (Fig 2, S1 Table). One mine was a spatial outlier and was excluded from spatial analyses. This mine was sited at a pre-existing gravel pit and was the only mine located beyond the boundary of quartzite sand deposits according to state geological data [54]. I compiled location information of frac sand mining activity from two databases, one compiled by the Wisconsin Center for Investigative Journalism [55] and the other by the West Central Regional Planning Commission in partnership with the Wisconsin Department of Transportation and the Mississippi Regional Planning Commission [56]. Both these efforts involved information gathered from county staff and other local and state resources. I checked mine locations listed in these databases against the most recent satellite images provided by Google Earth. Dates for these images ranged from 2009 to 2013. In cases where the mines were newer than the newest image date, and in all cases where mines were permitted but not yet in development, I plotted locations at least as precise as municipality, and in most cases as precise as a street address (both were often available in the above sources).

### Maximum entropy modeling for predicting probability of frac sand mine occurrence

Major considerations of any spatial analysis include defining the environment in which spatial patterns are to be analyzed, and accounting for heterogeneity within that environment [58]. It was important that the spatial analysis of mine pattern take into account that not all land containing silica sand was equally likely to be mined. Sand buried deeply underground [50] is less accessible than shallow sand and presents higher extraction costs. Mining is less likely to occur under certain land covers, e.g., wetlands and impervious surfaces; 65% of the study mines were located in agricultural areas, and 26% in forested areas [59]. Access to appropriate transportation infrastructure is also expected to influence facility siting [16]. Frac sand transport relies on roads that can handle heavy traffic of large trucks, and rail shipping of sand to hydrofracking sites in other parts of the country. Because these accessibility factors vary spatially, they are useful in differentiating silica sand deposits more and less likely to be mined.

I used a maximum entropy modeling technique to map frac sand mining probability within areas containing industrial quality silica sand [50]. Maximum entropy modeling has many applications in computer science [60] and is used in ecology for predicting species distributions based on presence-only data [57]. The explicitly spatial applications in ecology are of use in this study and guided software choice for this analysis. I chose Phillips, Anderson and Schapire’s [57] Maxent software for its ease of use and mapping capabilities. This software generates generalized linear models for predicting the probability of binary outcomes (e.g., mine presence or absence) across spatial extent defined by the user.

Inputs to Maxent included a location file with coordinates of existing and permitted frac sand mines, as well as four environmental datasets: sand depth [54], distance to major road [61], distance to rail [61], and land cover from the 2006 National Land Cover Data Set [59]. I prepared the environmental raster datasets in ArcGIS by clipping them so that they covered the same study region, and converted them to ascii files for input into the Maxent program. Using binomial logistic regression, Maxent produced a spatial probability surface—a raster in which each cell’s value is the probability of a frac sand mine occurring based on actual mine locations (a presence/absence surface serving as the dependent variable) and the values contained in the environmental datasets (independent variables). This output raster map had 30-m cell resolution, corresponding to the resolution of the input files.

Model performance can be measured by splitting data into two sets, one set used to train the model and one set used to test model performance [62,63]. Model fit for the test data can then be compared to fit for the training data using area under the curve (AUC) values calculated from receiver operating curves (ROCs) [63]. The higher the model’s AUC for the test data relative to the training data, the higher the model’s predictive power (see [62] for more information on the use of AUC in Maxent). A model that does no better than random prediction has an AUC value of 0.5. I set aside 25% of data cells as test data, and ROC curves show good predictive power of the model; model fit of test data (AUC = 0.747) approached that of training data (AUC = 0.809) (S1 Fig). A jackknife analysis in which multiple models are fit with and without each variable allows for a comparison of relative contribution of each variable on model fit [64]. Of the four variables, depth to sand was the most important predictor of mine presence, followed by land cover (S2 Fig). Distance to road and rail added relatively little predictive power to the model, likely because of the ubiquity of road and rail in much of the study area.

### Neutral landscape models for comparing frac sand mine distribution to null distributions

I addressed the question of whether frac sand mines were more prevalent in unzoned jurisdictions than would be expected based on location of suitable frac sand alone with three neutral landscape models at three different spatial extents. Neutral landscape models were introduced in the field of landscape ecology to resolve issues of non-replicability of landscapes [65–67]. By producing replicate “landscapes” absent some or all of the features of true landscapes, researchers are able to create null or baseline scenarios from which to compare real landscape configurations and test hypotheses [66,67]. The use of neutral models can help tease out the effects of geography, human activities and other factors on landscape pattern [67]. As in this study, they can also be used to determine if patterns of clustering exist in spatial data.

Determining if a set of point observations is spatially clustered commonly involves comparing the average nearest neighbor distance or Ripley’s K [23,68] of the observed points to that of randomly distributed points. The null hypothesis in that case would be that the points are randomly distributed in space. However, since the location of silica sand is in no way random, such approaches would not garner useful information in this study and results would be expected to show significant clustering of mines in all cases. Instead, I used the Maxent probability surface to construct sets of randomized points (“permutations”) that took into account the location of silica sand more and less likely to be mined. Areas with higher probabilities of silica sand mining were more likely to receive points than areas with lower probabilities. Permutations were created using the “Create Spatially Balanced Points” tool in ArcGIS 10.1 and Arcpy.

Running analyses at three different spatial extents allowed for examination of patterns at multiple scales, from global (statewide) to local (within-county). The three spatial extents of the neutral models were 1) all the silica sand areas in Wisconsin, 2) the silica sand areas in all of the 17 counties where actual mines were present, and 3) silica sand areas within each of these 17 counties separately. For each of the two multi-county neutral models, I used Arcpy to generate 500 permutations containing 101 points each, corresponding to the number of actual frac sand mines in the state. For the individual county neutral models, I generated 500 spatially balanced point layers for each county, each containing a number of points corresponding to the number of mines actually located in that county. From each set of 500 permutations I calculated confidence intervals from which to compare counts of actual mines in zoned and unzoned areas and those with and without land-use planning.

Zoning and planning data were compiled from a survey done by the Wisconsin Department of Administration in 2011 [51]. The survey indicated whether each township in the study area was under county, township, or no zoning, and whether it was in the process of or had previously undertaken a land-use planning process. Though land-use plans do not hold any regulatory authority, they offer a guiding vision for a community’s land-use management and provide an indicator of community involvement in land-use issues. To be certain townships considered zoned had zoning in place before 2009 when the first frac sand mines were established, I checked dates on zoning documents and made phone calls to government officials when date of zoning adoption was unclear. Due to my focus on zoning and the lack of any frac sand mining on public lands in Wisconsin, public lands were excluded from these analyses.

In this study, mine counts predicted by the neutral models represented what would be expected based only on geological suitability and proximity to transportation infrastructure, without consideration of any socio-political elements. In other words, the neutral models provided apolitical scenarios from which to compare actual mine prevalence. If geological suitability and proximity to transportation infrastructure are the most important drivers of mine siting decisions, mine counts will be well predicted by the neutral landscape models. If not, the effects of zoning and other socio-political and economic drivers should be investigated.

### Regression to determine the influence of socioeconomic status on mine prevalence

I chose variables for the regression models that would allow some insight into factors that may account for higher prevalence of mines in certain townships than others. I compiled socioeconomic data [69], tax data [70], and zoning data [51] at the township level for all townships containing at least one permitted or operating frac sand mine (n = 54) and a sample of townships without mines (n = 258). Unmined townships were selected if they were located in the same counties as a mined township and had at least 0.4 km^2^ of shallow silica sand (which corresponds to the low end of the range in the mined group). Silica sand was considered shallow if it was at a depth less than 15.25 m [50].

I fit models containing different combinations of the following variables: per capita income [69] to measure socioeconomic status; Euclidean distance to the boundary of the nearest U.S. Census Urbanized Area (defined as jurisdictions or clusters of jurisdictions with population > 250,000) to measure remoteness [71]; percent of the population white [69] to measure racial homogeneity; the average effective property tax rate [70], land value per km^2^ [72] and average parcel size in the township [72], measures of location cost; zoning status (zoned or unzoned) [51], a control variable for the area of shallow silica sand in the township [54]; and a county variable to control for county effects (the 312 townships were grouped into 17 counties). Dates of data sources reflect conditions as near as possible to conditions pre-2009 when the frac sand boom began. Demographic variables compiled from the U.S. Census Bureau’s decadal census were from 2000 because using data from the 2010 census could confound results in townships where mines were already present in 2010.

I examined relationships between socioeconomic variables and frac sand mine prevalence with generalized linear models (GLMs) using the negative binomial specification for count data in R [73]. The negative binomial specification was chosen over the simpler Poisson distribution because frac sand mine counts were slightly overdispersed (overdispersion parameter = 1.33 compared to the Poisson default of 1, p = 0.0004). Several combinations of variables had high Pearson correlation coefficients (income versus parcel size (-0.41) and land value (0.55); land value versus parcel size (-0.63) and tax rate (-0.41)). Because multi-collinearity can greatly impact standard errors, I calculated a variance inflation factor (VIF) for each variable included in the model [74]. If the square root of VIF is 2, the standard error is double what it would be if that variable were truly independent. Values greater than 2 or 3 may raise alarm in some cases [75].

Fitting multiple models served three purposes: to compare fit among models, to minimize multi-collinearity issues by putting highly correlated variables in separate models, and to see how stable each variable’s regression coefficient was in the presence of different combinations of other variables. Model 1 included only per capita income and the sand area control variable. Model 2 included only land value and the sand area control variable. Model 3 included all covariates except land value and tax rate, and Model 4 included all variables. All square roots of VIF for Models 3 and 4 were below 2, indicating that multi-collinearity was not severely influencing standard errors. Model 3 had lower VIF values and a lower AIC compared to Model 4, and a likelihood ratio test showed it to fit the data just as well (X^2^ = 0.20, p = 0.90). Model 3 estimates were used to predict mine counts at different distances from urbanized area while holding all other variables constant at their means.

## Results

### Frac sand mines clustered in unzoned areas

**Fig 3:**
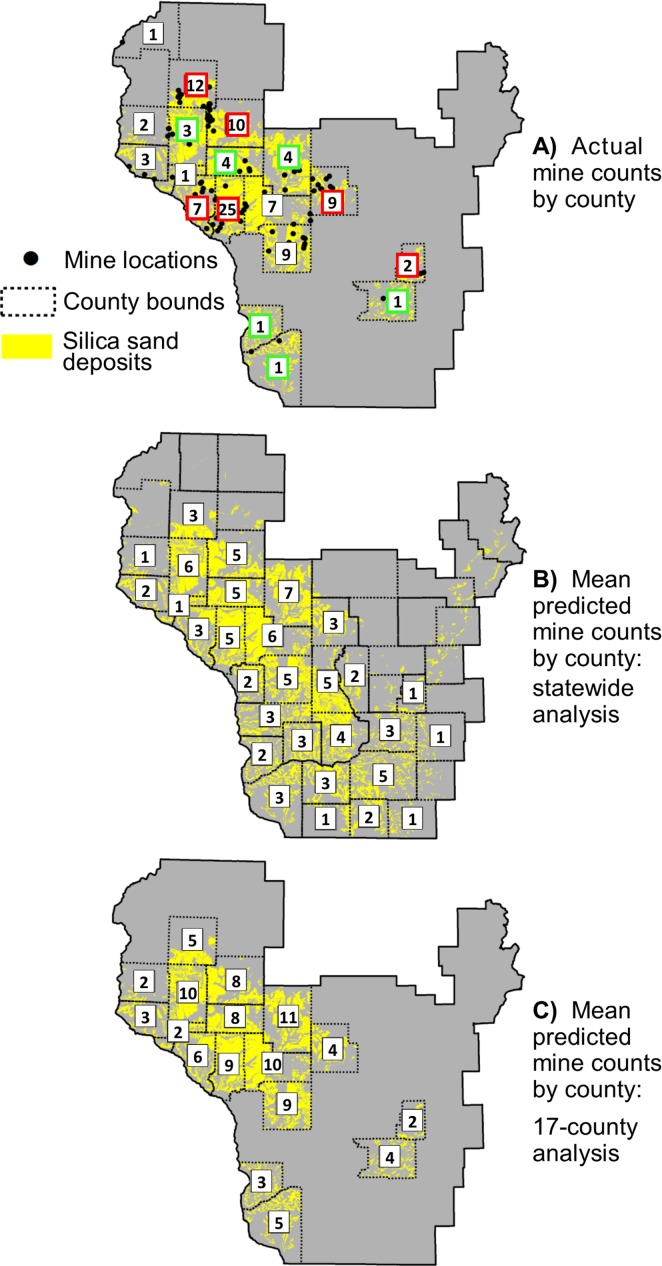
Actual (A) and predicted (B and C) frac sand mine counts per county. Predicted mine counts were based on two neutral landscape models at different spatial scales. Each predicted count is the average of 500 point permutations distributed based on probability of mining. Numbers outlined in red indicate actual mine counts that were higher than predicted confidence intervals in both neutral model analyses; numbers outlined in green indicate counts lower than predicted confidence intervals. Spatial extent (C) does not include the furthest northwest county because its mine was a spatial outlier.

**Fig 4:**
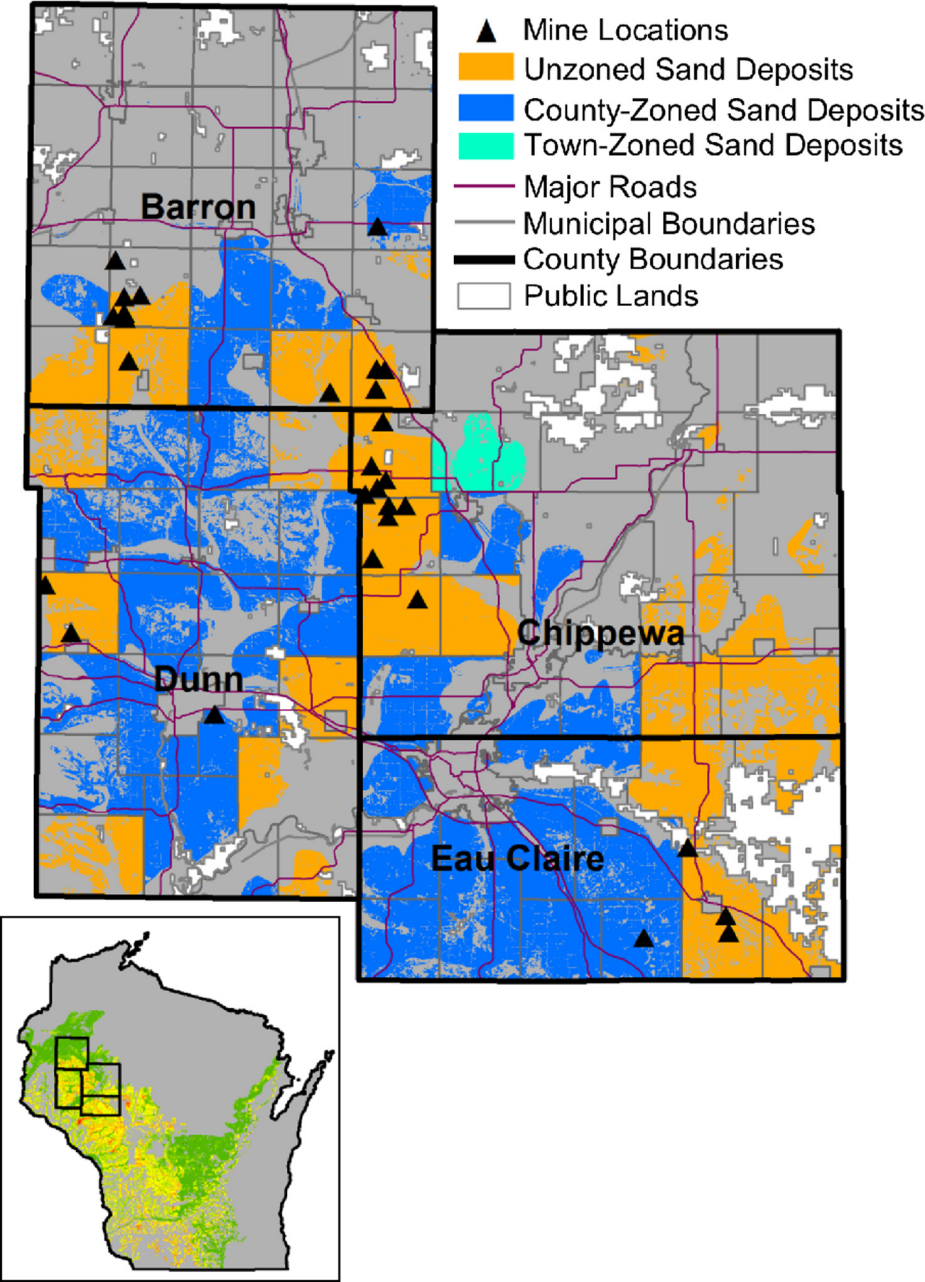
Four of the Wisconsin counties where frac sand mines clustered in unzoned jurisdictions. In seven of the nine counties having some unzoned land and at least three frac sand mines, mines were more concentrated in unzoned areas than would be expected due to geological suitability and transportation proximity alone.

Both multi-county analyses showed frac sand mines to be more concentrated in unzoned jurisdictions than would be likely if mine location depended solely on geophysical suitability and transportation access (Table 1). Individual county analyses showed preferential mine siting in unzoned areas in all but one county with at least three mines and some unzoned land. The one exception was Jackson County whose mine count in county-zoned areas (5) was slightly higher than the predicted 95% confidence interval (4.1–4.4). 46 counties contained silica sand suitable for frac sand mining. Of these, the statewide neutral model predicted 29 to be likely to contain at least one mine, though in actuality mines have been permitted and/or developed in only 17 counties. The 12 counties that were predicted to have mines but did not were concentrated in the southern part of the state (Fig 3). This result points to the potential of unmined areas of the state that are not currently being mined, and also the tendency of mines to cluster where mining infrastructure (e.g. rail load-outs and processing plants) is already established. Counties like Dunn and Eau Claire had fewer mines overall than would be expected due to chance but the mines they did have were concentrated in unzoned townships (Fig 4). Barron and Chippewa had more mines overall than would be expected due to chance, and their mines were also concentrated in unzoned townships.

**Table 1:** Mine counts and confidence intervals for neutral model predictions, by zoning and planning status for statewide and county-level analyses.

	Actual mine count, total	Actual mine counts by zoning and planning status (confidence intervals for expected counts in parentheses)^a^				
		Unzoned, No Planning	Unzoned, Planning	Town Zoning, No Planning	Town Zoning, Planning	County Zoning, Planning
Multi-County Analyses						
17 Counties	101	**18** (14–15)	**26** (20–21)	3 (2.8–3.1)	2 (6.8–7.4)	52 (55–56)
Statewide	101	**18** (11–12)	**26** (17–18)	**3** (2.4–2.8)	2 (12–12)	52 (58–59)
Individual County Analyses						
Trempealeau	25	—	—	—	—	25
Barron	12	**7** (2.8–3.1)	**4** (2.3–2.6)	0 (0.0–0.0)	0 (0.2–0.3)	1 (6.2–6.6)
Chippewa	10	0 (1.2–1.4)	**10** (4.8–5.2)	—	0 (0.9–1.1)	0 (2.6–2.9)
Wood	9	**5** (4.3–4.6)	**1** (0.4–0.5)	**3** (0.7–0.8)	0 (3.2–3.5)	—
Monroe	9	2 (1.8–2.1)	**3** (1.7–1.9)	—	0 (0.1–0.2)	4 (4.9–5.2)
Buffalo	7	—	—	—	—	7
Jackson	7	—	2 (2.6–2.8)	—	0 (0.0–0.1)	**5** (4.1–4.4)
Eau Claire	4	0 (0.1–0.2)	**3** (0.9–1.0)	—	0 (0.1–0.2)	1 (2.6–2.8)
Clark	4	**3** (2.3–2.5)	**1** (0.4–0.6)	0 (0.8–1.0)	0 (0.1–0.2)	—
Dunn	3	—	**2** (0.8–1.0)	0 (0.0–0.1)	0 (0.0–0.1)	1 (1.9–2.1)
Pierce	3	—	—	—	0 (0.4–0.6)	**3** (2.4–2.6)
Green Lake	2	0 (0.2–0.3)	0 (0.4–0.5)	—	0 (0.0–0.1)	**2** (1.2–1.3)
Saint Croix	2	—	0 (0.1–0.1)	0 (0.0–0.0)	0 (0.1–0.2)	**2** (1.7–1.8)
Pepin	1	0 (0.0–0.0)	0 (0.4–0.5)	—	**1** (0.5–0.6)	—
Crawford	1	0 (0.1–0.2)	0 (0.3–0.4)	—	**1** (0.4–0.5)	0 (0.1–0.2)
Columbia	1	—	0 (0.0–0.1)	0 (0.0–0.0)	0 (0.0–0.1)	**1** (0.9–0.9)
Grant	1	**1** (0.1–0.1)	0 (0.3–0.4)	—	0 (0.0–0.0)	0 (0.5–0.6)

^a^Value ranges in parentheses reflect the lower and upper limits of the 95% confidence interval predicted by the neutral model for each spatial extent. The actual mine count is in bold font if it is greater than the upper limit of the confidence interval. Dashes indicate an absence of silica sand present in a certain zoning category.

Though unzoned areas generally had higher mine counts than expected by the neutral models, two counties completely under countywide zoning ordinances contained more mines than expected based on the 17-county and statewide analyses: Buffalo (with 7 mines) and Trempealeau (25 mines). This result shows preferential mine siting did not occur only in unzoned jurisdictions. Looking further into this phenomenon, I compared neutral model results to the stringency of county land-use plans and zoning ordinances using data compiled from previous content analyses [76–78]. Both Trempealeau and Buffalo counties ranked low on a combined planning/zoning stringency score, even though Trempealeau’s zoning ordinance specifies 7 standards for nonmetallic mining (Table 2). However, there was insufficient power to detect an association between zoning/planning stringency and mine prevalence across all 14 counties with county zoning. The group of counties having higher-than-expected mine counts and the group having lower-than-expected mine counts exhibited non-significant differences (at the 0.05 level) in number of zoning standards for nonmetallic mining (p = 0.09), county land-use plan stringency (p = 0.75), and combined planning/zoning stringency score (p = 0.90) based on Mann Whitney test results.

**Table 2:** Stringency of county zoning ordinances and land-use plans in counties with higher- and lower-than-expected mine counts in county-zoned areas.

County name	Mine count in county-zoned land	Mine count in county-zoned land higher or lower than expected^a^	Number of nonmetallic mining standards in county zoning ordinance^b^	Land-use plan score^c^	Combined planning/zoning stringency score^d^
Trempealeau	25	higher	7	0.5	3.8
Buffalo	7	higher	0	0	0
Jackson	5	higher	14	4.5	13.3
Pierce	3	higher	10	3.5	10
Green Lake	2	higher	2	0	0.8
Saint Croix	2	higher	8	0	3.3
Columbia	1	higher	14	5	14.2
Monroe	4	lower	0	1.5	2.5
Barron	1	lower	7	2.5	7.1
Eau Claire	1	lower	8	3.5	9.2
Dunn	1	lower	0	2.5	4.2
Chippewa	0	lower	3	4.5	8.8
Crawford	0	lower	2	0	3.3
Grant	0	lower	0	2	3.3

^a^Based on multi-county neutral model analyses for counties that were entirely under county zoning, Trempealeau and Buffalo (Fig 3), and within-county neutral model analyses for all other counties (Table 1).

^b^From [76]. Standards include those either required or considered during the conditional use permitting process, as named in the county’s zoning ordinance [76] (see S1 Appendix for full list of standards).

^c^From [77]. Land-use plans were scored based on mentions of nonmetallic mining, ordinances and policies, and goals, as well as language strength [77] (See S1 Appendix for score equation).

^d^From [78]. Number of nonmetallic mining standards^b^ and land-use plan score^c^ were normalized and added to get a combined stringency score [78] (See S1 Appendix for detailed methods).

### Zoning better predicted mine prevalence than did socioeconomic factors

**Fig 5:**
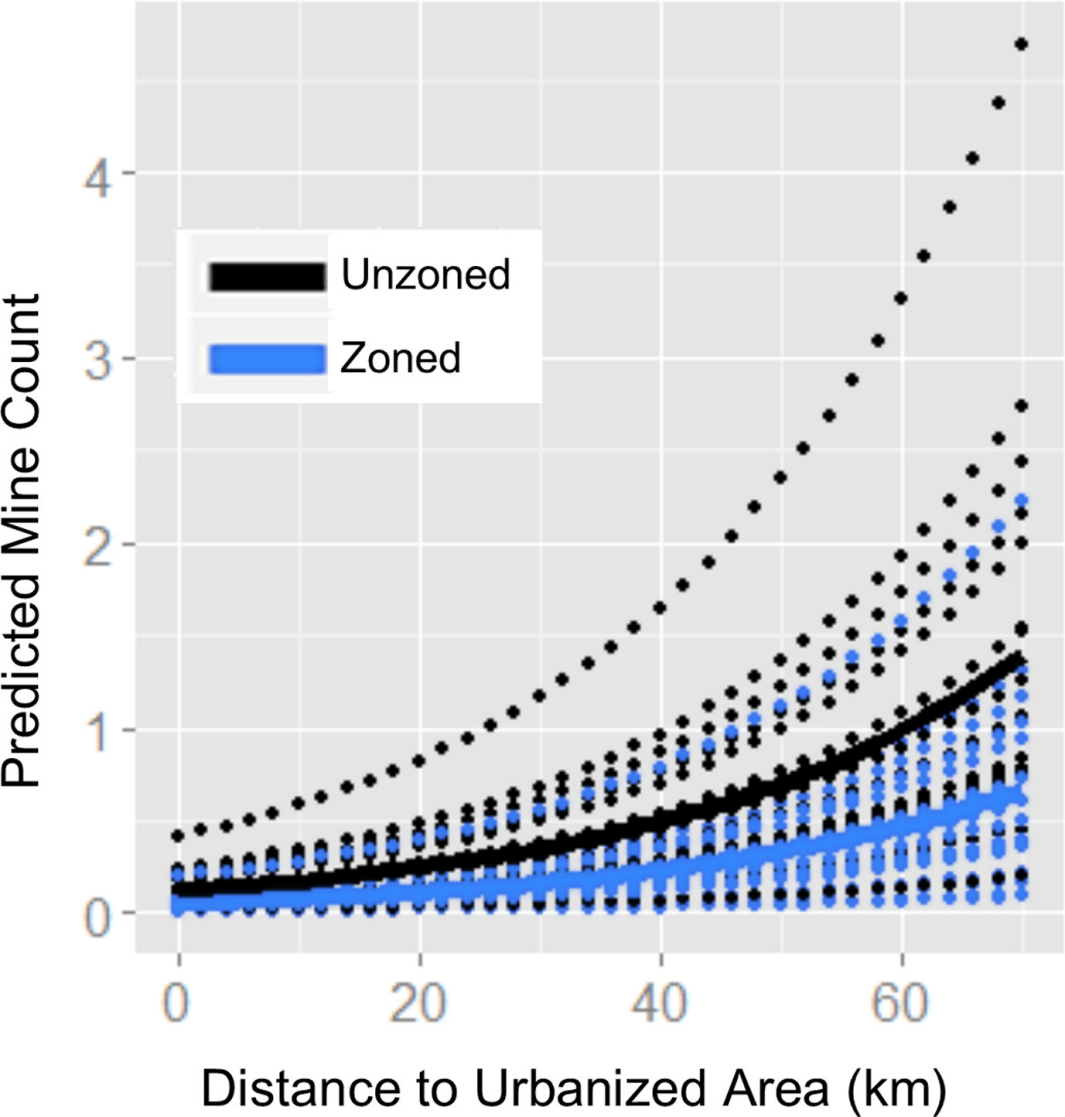
Predicted frac sand mine counts per Wisconsin township, plotted against distance to urbanized area. Mine count estimates were calculated using a generalized linear model (Model 3). Dotted lines show separate predictions for 17 Wisconsin counties; solid lines show the mean prediction across all counties, by zoning status.

Regression results showed remoteness (distance to urbanized area) to be positively associated with mine count, but showed no significant correlation between socioeconomic status or racial homogeneity and the frequency of frac sand mine sitings (Table 3). The latter results were likely due to the small amount of socioeconomic variation across townships regardless of mine count (S2 Table). Holding all other variables constant, the expected log count of mines in unzoned townships was 0.75 higher than the expected log count of mines in zoned townships, meaning the predicted mine count in unzoned townships was more than double that in zoned townships (exp(0.75) = 2.12). This result was consistent with the neutral model analyses showing higher-than-expected mine counts in unzoned areas. Predictions showed the variability in expected mine count across counties as well as the overall trend of more expected mines in unzoned areas (Fig 5). Zoned townships were on average wealthier, closer to urban areas, and had smaller areas of shallow sand (S3 Table), but even when controlling for these factors zoning was a significant predictor of mine count (Models 3 and 4). Per capita income and land value were not significant predictors of mine prevalence even in models that did not include zoning. Income was not a significant predictor when zoning was removed from Model 3 (coef = -0.059, SE = 0.0530, p = 0.27), and land value was not a significant predictor when it was subsequently switched with income in that model (coef = -0.00015, SE = 0.00050, p = 0.76).

**Table 3:** Relative log count of frac sand mines in Wisconsin townships as predicted by four generalized linear models.

Variable	Model 1 Coef. (SE)	Model 2 Coef. (SE)	Model 3 Coef. (SE)	Model 4 Coef. (SE)	√VIF, Model 3	√VIF, Model 4
(Intercept)	-1.79* (0.83)	-2.23** (0.32)	-4.30 (6.65)	-4.17 (6.78)	—	—
Per Capita Income ($1000s)	-0.029 (0.043)	—	-0.024 (0.054)	-0.018 (0.058)	1.29	1.35
Area of Shallow Silica Sand (km^2^)	**0.018** ** (0.004)	**0.017** ** (0.004)	**0.018** ** (0.005)	**0.018** ** (0.005)	1.34	1.36
Land Value ($1000s per km^2^)	—	-0.00023 (0.0004)	—	-0.00019 (0.0006)	—	1.45
Property Tax Rate (x100)	—	—	-1.32 (1.01)	-1.30 (1.07)	1.57	1.64
Average Parcel Size (ha)	—	—	—	-0.093 (0.20)	—	1.38
Distance to Urbanized Area (km)	—	—	**0.035** * (0.014)	**0.036** * (0.015)	1.70	1.78
% Population White	—	—	0.040 (0.063)	0.042 (0.064)	1.15	1.15
No Zoning	—	—	**0.75** * (0.38)	**0.75** * (0.38)	1.30	1.31
County (n = 17)	—	—	not shown	not shown	all < 1.81	all < 1.89
Residual df	309	309	289	287		
Residual deviance	158.6	159.1	155.8	156.1		
2 x Log Likelihood	-395.4	-395.5	-356.3	-356.1		
AIC	403.4	403.5	404.3	408.1		

*p<0.05,

**p<0.001

## Discussion

Spatial analyses provided evidence of preferential siting of frac sand mines in areas lacking zoning regulations, while regression analyses showed zoning to be a better predictor of mine prevalence than socioeconomic factors like per capita income, tax rate and land value. Two multi-county neutral model analyses and seven of eight within-county analyses showed significant clustering of mines in unzoned areas, supporting prior findings that industrial facilities are preferentially sited in jurisdictions with fewer regulatory impediments to siting and operations [9–13]. Higher-than-expected prevalence of frac sand mines in two counties under a countywide zoning ordinances also suggested that locations with homogenous rules are in some cases preferentially sited over locations with patchwork regulatory patterns. These two counties had low planning/zoning stringency scores [78], but there was no consistent link across counties between county-level zoning/planning stringency and mine prevalence on county-zoned land. Consistent with Fitchen’s [5] hypothesis, regression results showed mine prevalence to be positively correlated with remoteness, but contrary to this hypothesis and consistent with Bohon and Humphrey’s [3] findings, siting was not associated with socioeconomic status. Partially because the study was geographically constrained to areas containing silica sand, per capita income and racial homogeneity varied little between townships containing mines and those without mines. Though it has been argued that zoning’s effects are negated by market forces and zoning decisions favoring development [79], this study shows that presence/absence of zoning can be an important factor determining rural land-use change patterns.

Is the preferential siting of frac sand mines in unzoned jurisdictions due to deliberate actions of mining companies or are unzoned jurisdictions preferentially pursuing mines? This study alone does not answer this question, but prior ethnographic research suggests that frac sand mine siting is a product of aggressive propositioning by mining companies paired with favorable actions by local officials and residents who stand to gain financially [37]. Trepidation over these dealings from the community at large manifests itself in lawsuits, grassroots organizing and ad-hoc policymaking [80–82]. In Trempealeau County where the frac sand mining boom has been most intense, a unanimous vote passed a 12-month moratorium on new mining activity to allow time for health and safety concerns to be studied [80]. Several cities in that county have attempted land annexations to include mines in their boundaries with financial incentives at stake, actions that in some cases have led to legal challenges [81]. Long-term policy effects, the actions of cities, and land annexations to acquire mines are all fascinating avenues for further research.

The presence of zoning does not necessarily preclude mining. Even in districts zoned for non-industrial uses, the zoning ordinance may grant conditional use permits for LULUs like frac sand mines [76], or allow for rezoning of non-industrial land. This presents a major argument against zoning, that since it is subject to change it has little effect on development patterns in the face of market pressures [79]. However, because most frac sand lies in agricultural or forested areas and not industrial zones, and because conditional use permitting and rezonings can be contentious, public processes required by zoning law may provide hurdles for companies looking to mine. Conditional use permitting and rezonings requires public notification and a committee vote at minimum, and municipalities or counties can require a list of standards before a mining permit is granted [76]. During this process neighbors of the proposed site have an opportunity to mobilize against the action, and lengthy periods of contentious board meetings and public hearings can follow [37]. This process lies in stark contrast to that in unzoned townships where mining companies can deal privately with landowners and might purchase large tracts of farmland without notifying nearby landowners. A public meeting may occur before mining begins, but an unzoned township can typically approve the mine quickly with little to no legal standing for those who oppose the decision. The potential for lengthy and contentious public debates may provide a disincentive for mine sitings in zoned jurisdictions, and may partly explain the significant dampening effect of zoning on frac sand mine prevalence found in this study.

There are alternative regulatory options for rural jurisdictions that do not require adopting a full zoning ordinance. Non-zoning policy options for local governments include temporary moratoria, agreements with individual companies, licensing ordinances to regulate certain operations, and in some cases, legislation arising from direct citizen action via petition [82]. Temporary moratoria on frac sand mine permitting can allow a municipality time to draft a zoning ordinance. Specific agreements between municipalities and mine operators offer piecemeal approaches to regulating mining activities in lieu of zoning. A growing number of townships are adopting nonmetallic mining ordinances designed specifically to regulate frac sand mining (Wisconsin lawyers, personal communications). Of the 15 township ordinances on which I have compiled information (though in the absence of comprehensive records this number is an underestimate), 10 do not yet have permitted mines and are adopting such ordinances preemptively, four are unzoned townships that already have up to five mines, and one is under a county zoning ordinance and has one mine. However, state legislation introduced in Wisconsin would limit the ability of local governments to adopt ordinances to regulate nonmetallic mining in favor of statewide regulations designed to be more straightforward for the frac sand mining industry [83]. This legislation has so far failed to win senate approval.

Reluctance to adopt township or county zoning is common in rural areas. Slow-growth rural areas are characterized by long-term relationships among neighbors where informal agreements decide land-use conversion decisions [7]. As rural areas undergo suburbanization and inflow of new residents, informal land-use agreements among neighbors become less effective, and residents become more reliant on and accepting of “rule-based controls capable of disciplining developers” [7]. However, frac sand mines have proliferated in areas without demographic or political signs of suburbanization. In this case we see truly rural communities, often without comprehensive land use regulations, caught up in shifting energy markets with distant origins. Participation in these new markets promises benefits for some and negative consequences for others, and can be extremely polarizing even in previously cohesive communities [37]. Furthermore, a homegrown, independent spirit is a point of pride for local governments. Even when residents perceive a new industry like frac sand mining as undesirable or threatening, townships would often prefer to manage the issue on their own rather than adopt existing county zoning ordinances (Wisconsin county official, personal communication). Frac sand mining in Wisconsin provides an example of a sudden land-use change phenomenon that can take rural, unzoned communities by surprise. Rural communities can use the case of frac sand mining as motivation to discuss and plan for sudden land-use predicaments rather than wait to grapple with unfamiliar legal processes during a period of intense conflict.

## Supporting Information

S1 Appendix Explanation of county planning/zoning stringency scores. Summary of methods used by Risse and Haines [76–78] to rank counties based on stringency of county zoning ordinances [76], comprehensive land-use plans [77], and the two combined [78] as they relate to nonmetallic mining.
(DOCX)

S1 Fig Receiver operator curves (ROCs) and area under the curve (AUC) values for model fit based on training and test data. Seventy-five percent of cells were used to train the model and 25% were set aside as test data. The difference between lines for test and training data indicate the model’s predictive power. Both models performed better than random chance in predicting frac sand mine presence based on distance to major roads and rail lines, depth to sand, and land cover type.
(TIF)

S2 Fig Maxent jackknife results showing importance of individual variables in calculating frac sand mining probability. The contribution of each variable included in the Maxent analysis to (A) regularized training gain, a measure of fit to the input training data, and (B) area under the curve, a measure of predictive power, is calculated by specifying Maxent probability models with and without each variable. The table (C) gives “Percent Contribution,” the increase in regularized gain to the contribution of each variable for each iteration of the training algorithm, and “Permutation Importance,” the drop in training AUC for each variable, normalized as percentages, after random permutation of presence and background data. Variables included were “nlcd,” a categorical indicating land cover type based on 2006 National Land Cover Dataset categories [59]; “rail,” a continuous variable indicating distance from rail line [61]; “road,” a continuous variable indicating distance from major road [61]; and “sand,” a categorical variable indicating sand depth from the surface (under 5 ft, between 5 ft and 15.25 m, between 15.25 and 30.5 m, and over 30.5 m) [54].
(TIF)

S1 Table UTM coordinates of frac sand mines in Wisconsin: operational, in development, or permitted as of 2013 (reference datum NAD 1983). (XLSX)

S2 Table Means and standard deviations of model variables for study townships, by mining status. (XLSX)

S3 Table Means and standard deviations of model variables for study townships, by zoning status. (XLSX)
