# Supplementary material for: Frac Sand Mines Are Preferentially Sited in Unzoned Rural Areas
Source: PLoS One. 2015 Jul 2;10(7):e0131386. doi: 10.1371/journal.pone.0131386 (PMC4489872; doi:10.1371/journal.pone.0131386)
Supplement: S1 Appendix — Summary of methods used by Risse and Haines [76–78] to rank counties based on stringency of county zoning ordinances [76], comprehensive land-use plans [77], and the two combined [78] as they relate to nonmetallic mining. (DOCX) [file pone.0131386.s001.docx]

S1 Appendix. Summary of methods used by Risse and Haines [75–77] to rank counties based on stringency of county zoning ordinances [75], comprehensive land-use plans [76], and the two combined [77] as they relate to nonmetallic mining.

Twenty-four standards on nonmetallic mining were found in a review of Wisconsin county zoning ordinances [75]: water quantity requirements, explosive storage, erosion control/prevention, structure placement, safety precautions list, operations plan, maximum size, and water quality/drainage, noise level, landscaping/screening, lighting, airblast prevention, hours of operation, dust, odor, test data, permit length, road setback, other setback, proximity to market, equipment list, topography alteration, roads used, fuel placement. These standards were tallied for each county if they were either listed as requirements of all nonmetallic mines in the zoning ordinance or listed as considerations for issuing conditional use permits.

County land-use plans were scored based on the following rules [76]:

- Discussion of nonmetallic mines: assigned 1 point if map present and nonmetallic mining discussed, assigned 0.5 if only discussion present, assigned 0 otherwise
- Ordinances: assigned 2 points for referencing statewide reclamation ordinance and county zoning ordinance, 1 point if either one referenced, 0 otherwise
- Goals: assigned 1 point if goals/objectives of plan addressed nonmetallic mining, 0 otherwise
- Policies: assigned 1 point if specific policy action planned to address goals/objectives, 0 otherwise
- Strength of language: designated as “strong” if strong language present (e.g., “the county will identify…” or “will ensure…”); “weak” if weak language used (e.g., “the county should” or “will work to promote”); or “moderate” if a mix of strong and weak language was used. This classification was used as a weight in tabulating the final scores. Goal and policy point values were multipled by 1.5 if they were stated in the plan using strong language, by 1.25 for moderate language, or by 1 for weak language.
- Scores were assigned to counties according to the following equation: D+O+[(G+P)*S] where D=discussion, O=ordinances, G=goals, P=policies, S=strength.

Combined planning/zoning stringency scores were calculated by normalizing the zoning and planning scores by dividing them by the maximum value possible (24 in the case of zoning and 6 in the case of planning), then multiplying both numbers by ten and adding them together [77]. This gives a combined stringency score between 0 and 20.
