# Supplementary figures and images for: Frac Sand Mines Are Preferentially Sited in Unzoned Rural Areas
Source: PLoS One. 2015 Jul 2;10(7):e0131386. doi: 10.1371/journal.pone.0131386 (PMC4489872; doi:10.1371/journal.pone.0131386)

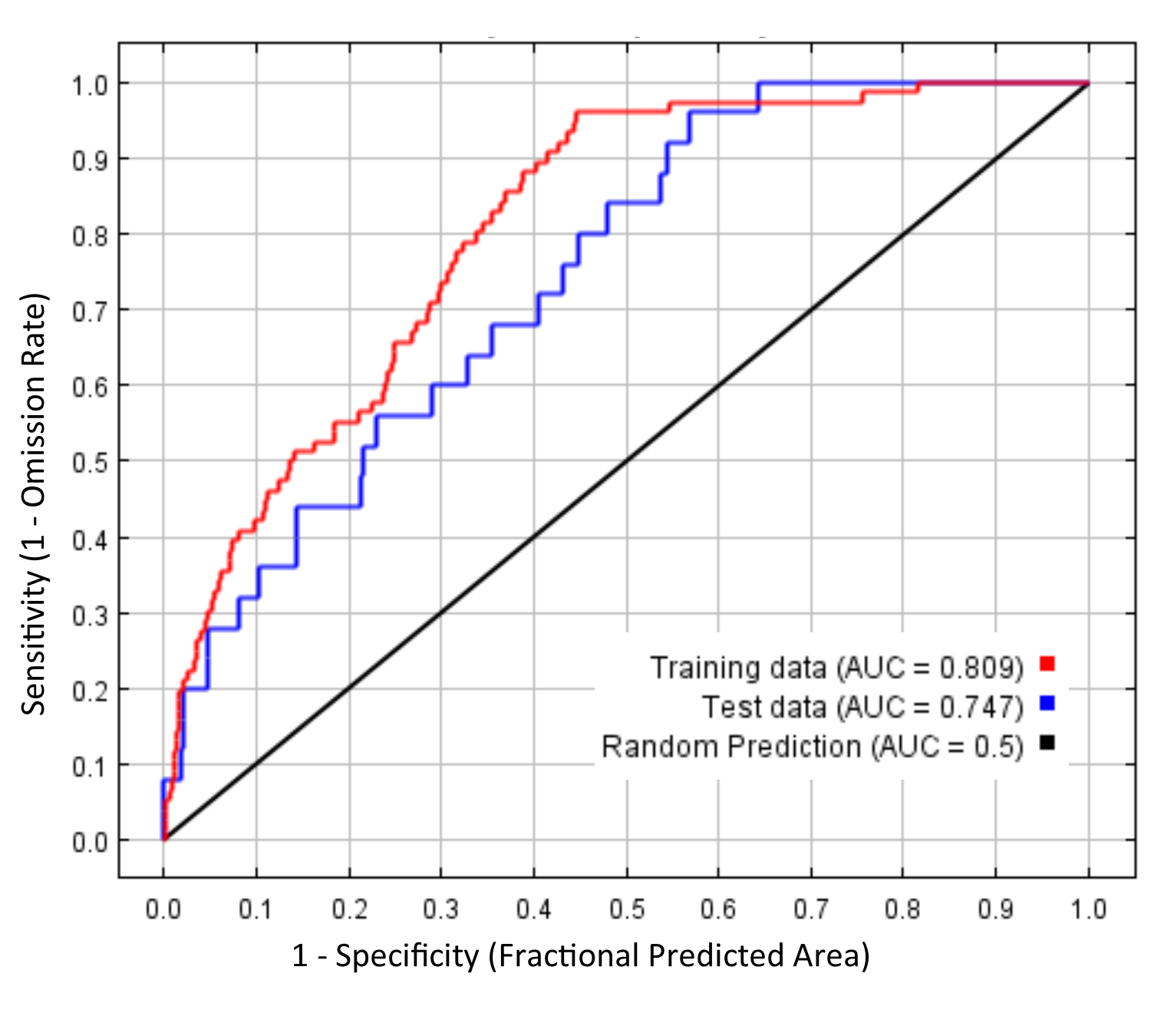

Supplement: S1 Fig — Seventy-five percent of cells were used to train the model and 25% were set aside as test data. The difference between lines for test and training data indicate the model’s predictive power. Both models performed better than random chance in predicting frac sand mine presence based on distance to major roads and rail lines, depth to sand, and land cover type. (TIF) [file pone.0131386.s002.tif]

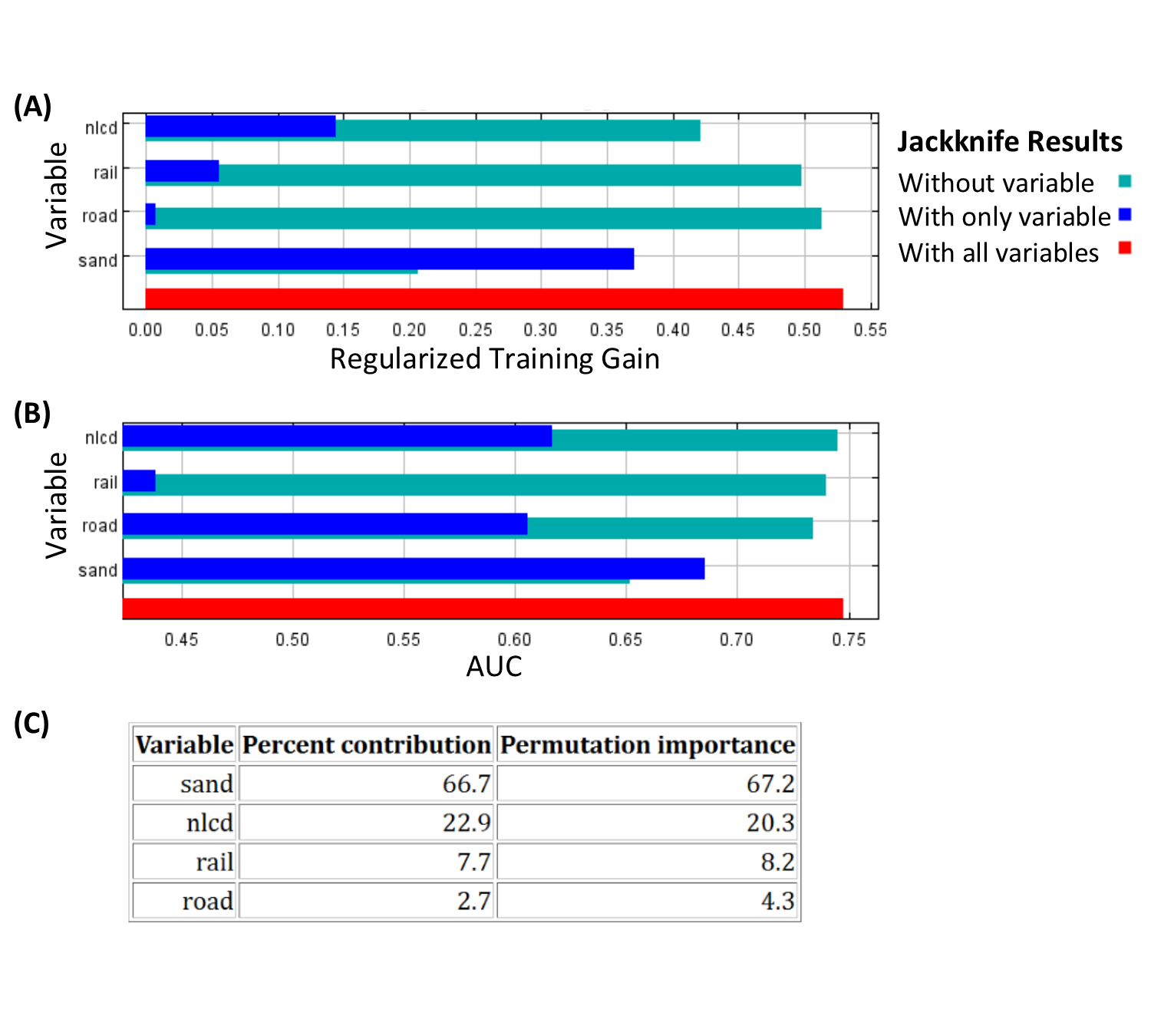

Supplement: S2 Fig — The contribution of each variable included in the Maxent analysis to (A) regularized training gain, a measure of fit to the input training data, and (B) area under the curve, a measure of predictive power, is calculated by specifying Maxent probability models with and without each variable. The table (C) gives “Percent Contribution,” the increase in regularized gain to the contribution of each variable for each iteration of the training algorithm, and “Permutation Importance,” the drop in training AUC for each variable, normalized as percentages, after random permutation of presence and background data. Variables included were “nlcd,” a categorical indicating land cover type based on 2006 National Land Cover Dataset categories [59]; “rail,” a continuous variable indicating distance from rail line [61]; “road,” a continuous variable indicating distance from major road [61]; and “sand,” a categorical variable indicating sand depth from the surface (under 5 ft, between 5 ft and 15.25 m, between 15.25 and 30.5 m, and over 30.5 m) [54]. (TIF) [file pone.0131386.s003.tif]
